# Reference Transcriptome Data in Silkworm *Bombyx mori*

**DOI:** 10.3390/insects12060519

**Published:** 2021-06-03

**Authors:** Kakeru Yokoi, Takuya Tsubota, Akiya Jouraku, Hideki Sezutsu, Hidemasa Bono

**Affiliations:** 1 Insect Genome Research and Engineering Unit, Division of Applied Genetics, Institute of Agrobiological Sciences (NIAS), National Agriculture and Food Research Organization (NARO), 1-2 Owashi, Tsukuba, Ibaraki 305-8634, Japan; joraku@affrc.go.jp; 2 Research Center for Agricultural Information Technology (RCAIT), National Agriculture and Food Research Organization (NARO), Kintetsu Kasumigaseki Building Kasumigaseki 3-5-1, Chiyoda-ku, Tokyo 100-0013, Japan; 3 Transgenic Silkworm Research Unit, Division of Biotechnology, Institute of Agrobiological Sciences (NIAS), National Agriculture and Food Research Organization (NARO), 1-2 Owashi, Tsukuba, Ibaraki 305-8634, Japan; tsubota@affrc.go.jp (T.T.); hsezutsu@affrc.go.jp (H.S.); 4 Database Center for Life Science (DBCLS), Joint Support-Center for Data Science Research, Research Organization of Information and Systems, 1111 Yata, Mishima, Shizuoka 411-8540, Japan; bonohu@hiroshima-u.ac.jp; 5 Program of Biomedical Science, Graduate School of Integrated Sciences for Life, Hiroshima University, 3-10-23 Kagamiyama, Higashi-Hiroshima City, Hiroshima 739-0046, Japan

**Keywords:** silkworm, *Bombyx mori*, transcriptome analysis, RNA-seq, gene model transcriptional factor

## Abstract

**Simple Summary:**

The silkworm *Bombyx mori* is a lepidopteran model insect with biological and industrial importance. Its high-quality genome sequence has recently become available and the utilization of this information, in combination with extensive transcriptomic analyses, is expected to provide an elaborate gene model. It will also be possible to clarify the gene expression in detail using this approach. In the present study, we established reference transcriptome data for the silkworm and performed a detailed examination of the gene expression in silkworm tissues. The results obtained will contribute to our understanding of silkworm biology and further promote the industrial application of the silkworm and other insects.

**Abstract:**

Herein, we performed RNA-seq analysis of ten major tissues/subparts of silkworm larvae. The sequences were mapped onto the reference genome assembly and the reference transcriptome data were successfully constructed. The reference data provided a nearly complete sequence for *sericin*-*1*, a major silk gene with a complex structure. We also markedly improved the gene model for other genes. The transcriptomic expression was investigated in each tissue and a number of transcripts were identified that were exclusively expressed in tissues such as the testis. Transcripts strongly expressed in the midgut formed tight genomic clusters, suggesting that they originated from tandem gene duplication. Transcriptional factor genes expressed in specific tissues or the silk gland subparts were also identified. We successfully constructed reference transcriptome data in the silkworm and found that a number of transcripts showed unique expression profiles. These results will facilitate basic studies on the silkworm and accelerate its applications, which will contribute to further advances in lepidopteran and entomological research as well as the practical use of these insects.

## 1. Introduction

The silkworm *Bombyx mori* is a lepidopteran insect that has been utilized in studies of physiology, genetics, molecular biology, and pathology. Functional analyses of genes related to hormone synthesis/degradation, pheromone reception, larval marking formation, and virus resistance have been performed using this silkworm [1,2,3,4,5], and the findings obtained have contributed to the promotion of insect science. The silkworm has the ability to produce large amounts of silk proteins, which is one of the most prominent characteristics of this species. Silk proteins are mainly composed of the fibrous protein Fibroin and aqueous protein sericin, which are produced in the larval tissue silk gland (SG) [6]. A transgenic technique has been applied to the silkworm [7] that has enabled the production of a large amount of recombinant proteins through the introduction of transgenes for overexpression in SG [8]. The silkworm can be utilized as a significant bioreactor through this approach.

Based on its biological and industrial importance, the whole genome sequence of the silkworm was reported in 2004 by two research groups [9,10]. This was the first lepidopteran genomic analysis and has served as a fundamental basis for genomic studies on Lepidoptera. This silkworm genome data were updated in 2008 [11], and related data have since become available, including microarray-based gene expression profiles, a BAC-based linkage map, and full-length cDNA data [12,13,14]. These data have strongly promoted studies on *B*. *mori* and other lepidopteran insects in the past few decades.

A new and high-quality reference genome assembly of the silkworm p50T (*daizo*) strain using PacBio long-read and Illumina short-read sequencers was reported in 2019 [15]. The new genome assembly consists of 696 scaffolds with N50 of 16.8 Mb and only 30 gaps. A predicted new gene model based on this novel genome assembly, using cDNA, protein, and RNA-seq data as hints, was constructed and was more precise that the previous model made via the old genome assembly [15]. The next step in the establishment of genome-related data is transcriptome data, which contain reference transcriptome sequence data and gene expression profiles in major tissues. These data will significantly contribute to advances in research on the silkworm and other lepidopterans.

**Figure 1:**
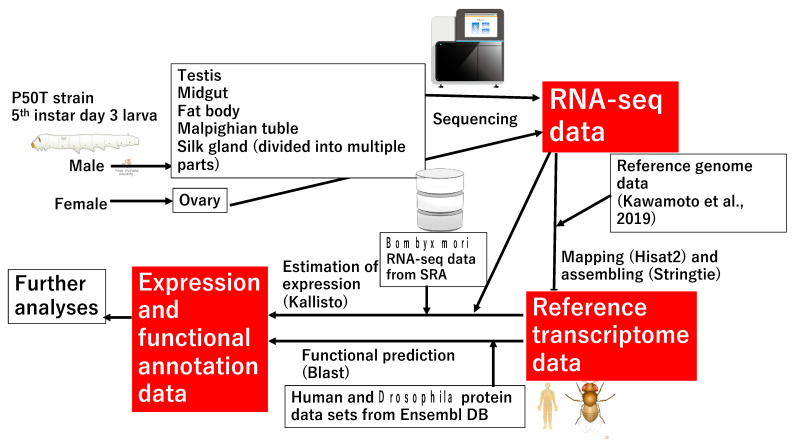
Workflow of the data analysis performed in the present study. To obtain reference transcriptome sequences, Fastq data of 10 tissues/subparts from fifth instar larvae were mapped to the new reference genome [15]. Kallisto software was used to estimate the expression abundance of each transcript in these tissues. Regarding RNA-seq data obtained from the public database (o751 strain; see Table 1). We performed a Blast search against human and *Drosophila* genome data to perform functional annotations of the reference transcriptome. The images in Figure 1 is from TogoTV (© 2016 DBCLS TogoTV).

In the present study, we constructed a reference transcriptome dataset using RNA-seq data obtained from ten major tissues/subparts of silkworm larvae (Figure 1). RNA-seq data were mapped on the new genome assembly and reference transcriptome sequence data were successfully constructed. We also performed functional annotation of the reference transcriptome using human and *Drosophila* protein datasets. in addition to NCBI-nr data. The established reference transcriptome sequence data provided a nearly complete structure for *sericin*-*1* (*Ser1*), a major silk gene with a complex sequence. The expression of the transcriptome was investigated in each tissue, and the expression of a number of transcripts was found to be confined to tissues such as the testis (TT). Among them, genes with transcripts that were strongly expressed in the midgut (MG) formed tight genomic clusters, suggesting that they originated via gene duplication. The transcripts of transcriptional factor (TF) genes expressed in specific tissues or SG subparts were also detected, and we speculate that these genes play key roles in the major biological process of these tissues/territories. The present results will accelerate molecular biological studies on the silkworm as well as other related species, and this is an essential milestone to promote entomological research as well as the practical use of insects.

## 2. Materials and Methods

### 2.1. Silkworm Rearing, RNA Extraction, and Sequencing

The silkworm p50T strain was reared on an artificial diet (Nihon Nosan Kogyo, Yokohama, Japan) at 25 °C under a 12-h light/dark photoperiod. The SG, fat body (FB), MG, Malpighian tuble (MT), TT, and ovary (OV) were dissected on the third day of the fifth instar larvae. The SG was further subdivided into the anterior silk gland (ASG), anterior part of the middle silk gland (MSG_A), middle part of the middle silk gland (MSG_M), posterior part of the middle silk gland (MSG_P), and posterior silk gland (PSG). Each tissue/subpart was dissected from one individual, and three biological replicates were obtained and separately analyzed (see Table 1). The tissues were homogenized using ISOGEN (NIPPON GENE, Tokyo, Japan) and the SV Total RNA Isolation System (Promega, Madison, WI, Tara) was used for the RNA extraction. The total RNA samples extracted were sequenced by Illumina NovaSeq6000 (Macrogen Japan Corp., Kyoto, Japan).

### 2.2. Construction of RTD and Estimation of the Expression of Each Transcript

The raw RNA-seq data of 30 samples were trimmed by Trimmomatic version 0.36 [16]. The trimmed RNA-seq data of each tissue were mapped to the new reference genome with a new gene model using HISAT2 version 2.1.0 [15,17]. The mapped data were each assembled to transcriptome data by StringTie version 1.3.3 [18]. The 30 transcriptome datasets were merged into one transcriptome dataset, referred to as “a reference transcriptome” by StringTie. GffCompare version 0.10.6 was used (URL: https://ccb.jhu.edu/software/stringtie/gffcompare.shtml: Accessed on 1 June 2021) for comparisons with the reference transcriptome and previously reported gene sets [15]. The transcripts detected at the newly identified loci were categorized into the “new loci” group, those newly detected at the previously identified loci into the “new isoform” group, and other genes and transcripts into the “identified in gene models”.

To estimate the expression of the reference transcriptome in 30 samples, the raw fastq data of each sample and the reference transcript data were used with Kallisto version 0.44.0 [19]. The raw RNA-seq data of multiple tissues in *B. mori* strain o751 from the Sequence Read Archive (SRA) and reference transcript data were used in comparisons of the transcriptome data; the accession numbers of the raw RNA-seq data are DRA005094, DRA005878, and DRA005094 [20,21,22].

We used TIBCO Spotfire Desktop (version 7.6.0) software with the “Better World” program license (TIBCO, Inc., Palo Alto, CA, USA; Tara, URL: http://spotfire.tibco.com/better-world-donation-program/: Accessed on 1 June 2021) for the classification of differentially expressed samples in silkworm tissues in HC using Ward’s method. Morpheus was also used for HC (https://software.broadinstitute.org/morpheus: Accessed on 1 June 2021). R (version 3.6.0) was used in the PCA analysis. Regarding the Venn diagram construction and the scatter plot analysis, R (version 4.0.2) was used. The relationships among the gene expression profiles in the SG territories were evaluated using Spearman’s rank correlation.

### 2.3. Annotation for the Reference Transcriptome and Functional Enrichment Analyses

Transcoder (version 5.5.0: URL: https://transdecoder.github.io/: Accessed on 1 June 2021) was used to identify the coding regions within the transcript sequences and to convert transcript sequences to amino acid sequences. Transcriptome sequence sets were compared at the predicted amino acid sequence level by the successive execution of the blastp program in the NCBI BLAST software package (version 2.9.0+), with default parameters and an e-value cut-off of 1e-10 [23]. Regarding the reference database sets to be blasted, human and fruit fly (*D. melanogaster*) protein datasets in the Ensembl database (version 97) were used because the sequences of these organisms were functionally well-annotated and amenable to computational methods, such as a pathway analysis [24]. The names of the top-hit genes in the human and fruit fly datasets were annotated to *B. mori* transcripts utilizing Ensembl Biomart [25] and Spotfire Desktop software under TIBCO Spotfire’s “Better World” program license. Functional enrichment analyses (FEA) were performed using the metascape portal site (version 3.5 on 10 May 2021) [26] with annotation results against the human gene set.

### 2.4. Comparison of Gene Structures among Different Models

The gene structures among the reference transcriptome data (RTD), gene model data (GMD), and cDNA-based data were compared in the silkBase (URL: http://silkbase.ab.a.u-tokyo.ac.jp/cgi-bin/index.cgi: Accessed on 1 June 2021) or KAIKObase [27]. Amino acid sequences were aligned using CLC Genomics Workbench 20.0.04 (QIAGEN, Aarhus, Denmark).

**Table 1:** Samples for RNA-seq and run accession IDs.

Sample	SRA Run ID	Strain	Sex	Reference
ASG-1,2,3	DRR186474,DRR186475,DRR186476	p50T	Male	This study
MSG_A-1,2,3	DRR186477,DRR186478,DRR186479	p50T	Male	This study
MSG_M-1,2,3	DRR186480,DRR186481,DRR186482	p50T	Male	This study
MSG_P-1,2,3	DRR186483,DRR186484,DRR186485	p50T	Male	This study
PSG-1,2,3	DRR186486,DRR186487,DRR186488	p50T	Male	This study
FB-1,2,3	DRR186489,DRR186490,DRR186491	p50T	Male	This study
MG-1,2,3	DRR186492,DRR186493,DRR186494	p50T	Male	This study
MT-1,2,3	DRR186495,DRR186496,DRR186497	p50T	Male	This study
TT-1,2,3	DRR186498,DRR186499,DRR186500	p50T	Male	This study
OV-1,2,3	DRR186501,DRR186502,DRR186503	p50T	Female	This study
BN_TT-1,2,3	DRR068893,DRR068894,DRR068895	o751	Male	[25]
BN_FB-1,2,3	DRR095105,DRR095106,DRR095107	o751	Male	[27]
BN_MG-1,2,3	DRR095108,DRR095109,DRR095110	o751	Male	[26]
BN_MT-1,2,3	DRR095111,DRR095112,DRR095113	o751	Male	[27]
BN_SG-1,2,3	DRR095114,DRR095115,DRR095116	o751	Male	[27]

### 2.5. RT-PCR

cDNA was synthesized using Superscript IV (Thermo Fisher Scientific Inc., Waltham, MA, USA) according to the manufacturer’s instructions. Five hundred nanograms of the total RNAs extracted from the ASG, MSG_A, MSG_M, MSG_P, and PSG were used for the cDNA synthesis. KOD FX neo polymerase (Toyobo, Osaka, Japan) was used for RT-PCR. The PCR conditions were as follows: 95 °C for 1 min, followed by 22 cycles (for rp49) or 30 cycles (for TF genes) of 95 °C for 30 s, 58 °C for 30 s, followed by 68 °C for 1 min, and additional 68 °C for 1 min after the cyclic phase. The primer sequences are listed in Table S1.

## 3. Results

### 3.1. Reference Transcriptome Data

**Figure 2:**
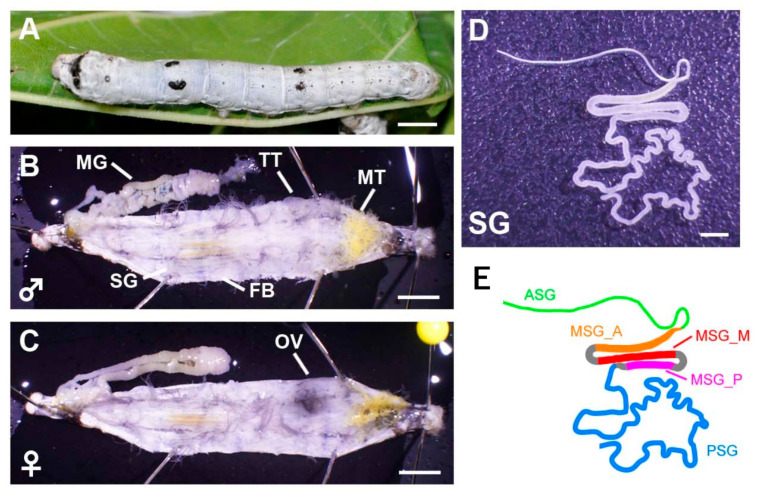
Tissues used in this study. (**A**) Final (fifth) instar larva of the silkworm p50T (daizo) strain. Scale bar = 5 mm. (**B**,**C**) Male (**B**) or (**C**) female individuals dissected on the third day of fifth instar larvae. Scale bar = 5 mm. MG—mid gut; TT—testis; MT—Malpighian tubules; SG—silk gland; FB—fat body; OV—ovary. (**D**,**E**) Image (**D**) and schematic (**E**) of the silk gland. ASG—anterior silk gland; MSG_A—anterior part of the middle silk gland; MSG_M—middle part of the middle silk gland; MSG_P—posterior part of the middle silk gland; PSG—posterior silk gland. Scale bar = 2.5 mm.

**Figure 3:**
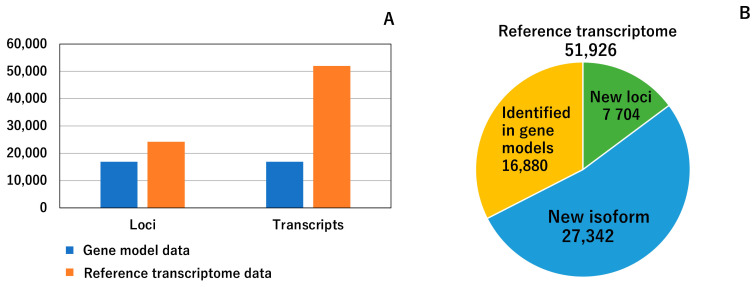
Basal characteristics of the reference transcriptome. (**A**) Comparison of the gene model data [15] and the reference transcriptome data of the present study. The numbers of the loci and transcripts are shown. These numbers were calculated from gff files of the two data sets. (**B**) Classification of 51,926 transcripts. Each transcript was classified into three categories, and the numbers of the three categories are shown in a pie chart. Definitions of the three categories are described in the main text.

We performed a transcriptomic analysis of the major silkworm larval tissues, namely, the SG, FB, MG, MT, TT, and OV, to acquire more expanded RNA-seq data (Figure 2 and Table 1). The gene expressions were clearly differentiated among subregions in the SG [28,29], and, thus, we subdivided the SG into five subparts and investigated the gene expression in each region (ASG, MSG_A, MSG_M, MSG_P, and PSG; Figure 2 and Table 1). In total, ten tissues/subparts were dissected from the fifth instar third day larvae of p50T strain with three biological replicates (Table 1). Thirty sets of RNAs were used for RNA-seq. We mapped the RNA-seq data on the reference genome assembly using information from the previously established gene model [15] (hereafter referred to as gene model data (GMD)) and constructed reference transcriptome data (RTD). Hereafter in this article, we defined “gene” and “transcript” as a representative sequence producing a protein of single or multiple isoforms in a single loci shown in GMD data and a isoform derived from a gene shown in RTD data. RTD comprises 51,926 transcripts in 24,236 loci (Figure 3A), the numbers of which are higher than those of GMD (24,236 vs. 16,845 loci and 51,926 vs. 16,880 transcripts; see Figure 3A,B). Therefore, RTD is an extension of GMD. To perform functional annotations, coding sequence (CDS) regions and amino acid sequence data were constructed using RTD (Data S1), and it was found that 39,619 transcripts, derived from 16,632 loci, had at least one CDS in RTD. The predicted amino acid sequences were used for gene functional annotations through a homology search against human and Drosophila gene sets. This analysis revealed that 26,698 transcripts showed homology to human transcripts and 29,177 to fruit fly transcripts (Data S2). We also performed a blastp analysis using the NCBI nr database and found that 43,358 amino acids had homological proteins in this database (Data S3).

### 3.2. Comparison between Constructed Reference Transcriptome Data and Previous Gene Model Data

RTD represents a marked improvement over GMD. Several misassembled genes are present in GMD, as represented by the KWMTBOMO00087-88, KWMTBOMO00196-197, or KWMTBOMO00222-223 genes [15]. These genes are split into two structures, even though full-length cDNA data define them as single genes: BMgn002111, BMgn000626, and BMgn000572 (full-length cDNA data ID) covers the KWMTBOMO00087-88, 00196-19,7 and 00222-223 gene regions, respectively (Figure S1) [14,15]. We investigated their structures in our model and found that all were accurately predicted (MSTRG.494.1, MSTRG.649.1-2, and MSTRG.704.1-3; Figure S1). The elucidated gene structure was attributed to the extensive RNA-seq analysis in the present study; the previous study lacked gene expression data from tissues such as TT, OV, MT, and PSG, and these genes were all strongly expressed in these tissues (Table S2).

RTD also provided a number of novel genes/isoforms. Comparisons of RTD with GMD revealed that among the 51,926 transcripts identified, 7704 belonged to the new loci group, whereas 27,342 were categorized as new isoforms (Figure 3B, see Materials and Methods). Among the 7704 new loci group transcripts, a number of transcripts were also present in the previously established full-length cDNA-based gene model [14]. However, 2324 transcripts did not hit this gene set and, thus, the genes to which these transcripts belong were perceived to be novel genes. An expression analysis revealed that many of these transcripts belonging to these genes were commonly expressed in all of the tissues investigated herein, whereas the other transcripts were exclusively expressed in specific tissues, such as TT (Figure S2). The functional annotation analysis revealed that newly identified transcripts included a trypsin inhibitor (MSTRG.14562.2), carboxypeptidase (MSTRG.16874.1-3), and pyruvate kinase (MSTRG.18651.2). Therefore, our RTD represents a significant improvement over GMD (Additional file 4).

### 3.3. Elaborated Structures of Silk Genes

Silk production is one of the most prominent characteristics of the silkworm. Silk genes are strongly expressed in the silk-producing tissue SG, and our extensive RNA-seq data are expected to show highly elaborated models for these genes. *Ser1* is one of the major silk genes, is strongly expressed in the MSG, and encodes a >400-kDa serine-rich protein [6,30]. *Ser1* is composed of nine exons, among which exon 6 has a long repetitive sequence with a length of ~6500 bp [6,31]. The full-length sequence of this exon is yet to be elucidated because of its complexity. We demonstrate herein that our model MSTRG.2477.1 provided an almost complete sequence for this exon (6234 bp; Figure S3). Exon 6 encodes serine, glycine, threonine, asparagine, and aspartic acid-rich residues (Figure S4), which is consistent with previous findings showing that *Ser1* comprises large numbers of these residues (Table S3) [32]. The detailed structural analysis revealed that the long repetitive motif identified here comprised 53 repeats of a 38-amino acid unit (Figure S5). Each unit had serine-rich residues and a slight difference was observed in the sequences among units (Figure S5) [6]. The 38-amino acid-based repeat unit was also observed in exon 8 of *Ser1* (Figures S4 and S5) or in the sericins of saturniid species [6,33], and, thus, the repeat unit of this length is expected to have a structural function in a number of sericin proteins.

We also observed significant improvements for other sericin genes. *Sericin-3* (*Ser3*) is another major silk protein that has a relatively soft texture and possesses serine-rich residues [34,35]. In GMD, a 73-bp deletion was detected in exon 3, and because of this structural error, a frame shift was present in the predicted amino acid sequence (KWMTBOMO06311; Figure S6). In contrast, our RTD (MSTRG.2595.1) successfully provided an accurate gene structure (Figure S6). *Sericin-4* (*Ser4*) is another sericin protein that is composed of 34 exons [36]. This gene is split into three distinct structures in GMD (KWMTBOMO06324, KWMTBOMO06325, and KWMTBOMO06326), whereas RTD provided an exact model (MSTRG.2610.1; Figure S7). Collectively, these results suggest that our RTD provided highly defined structures, even for complex silk genes.

### 3.4. Estimating the Abundance of the Reference Transcriptome in Multiple Tissues 

**Figure 4:**
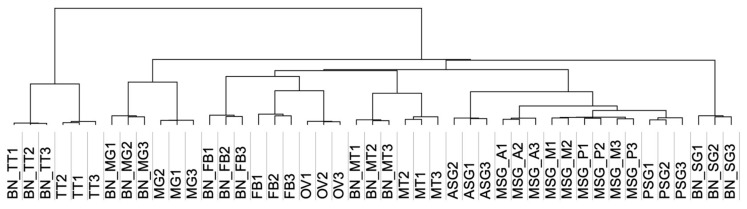
Hierarchical clustering of expression data in 45 samples of transcripts showing a tpm value > 30 in at least one sample. Abbreviations that start with “BN” indicate samples collected in a previous study [20,21,22]. The numbers added to the abbreviations mean biological replicates.

Our extensive transcriptomic analysis provided fundamental insights into the transcriptomic expression profiles in multiple silkworm tissues. The expression abundance of each transcript was calculated as transcripts per million (tpm; Additional file 14) and the transcriptomic expression was compared among tissues through two independent methods, one for hierarchical clustering (HC) and another for a principal component analysis (PCA). To avoid the effects of low expression transcripts, we performed these analyses using transcripts with tpm values > 30 in at least one sample. HC using all transcript tpm data were also performed for comparison. These analyses revealed that the biological replicate samples collected herein were highly reproducible, because they were derived from the same tissues forming tight clusters (Figure 4; Figure S8). In the HC analysis, a single cluster was formed for the MSG_M and MSG_P in each sample (Figure 4; Figure S8B), and we speculated that this was as a result of the highly conserved transcriptomic expression between these SG territories. A correlation analysis of the transcriptomic expression using SG transcriptome data supported this hypothesis (Table S4). A previous study performed an RNA-seq analysis of multiple silkworm larval tissues in another silkworm strain, o751 [20,21,22], and these RNA-seq data were added to our analysis (Figure 4, Table 1, and Figure S8; these samples are referred to as BN_MG, BN_FB, BN_MT, BN_SG, and BN_TT). We found that the samples collected from the same tissues clearly formed clusters (Figure 4). In the o751 strain, the SG was collected as a whole tissue. Although all of the SG subparts (MSG_A, MSG_M, MSG_P, ASG, and PSG) were closely located in the HC analysis of transcripts with >30 (tpm) in at least one sample, BN_SG formed a single cluster distant from those of the SG subparts (Figure 4). Therefore, our transcriptomic data are a robust platform for analyzing and comparing the gene expressions in multiple tissues.

### 3.5. Transcript Abundance in Each Tissue and in the Silk Gland

**Figure 5:**
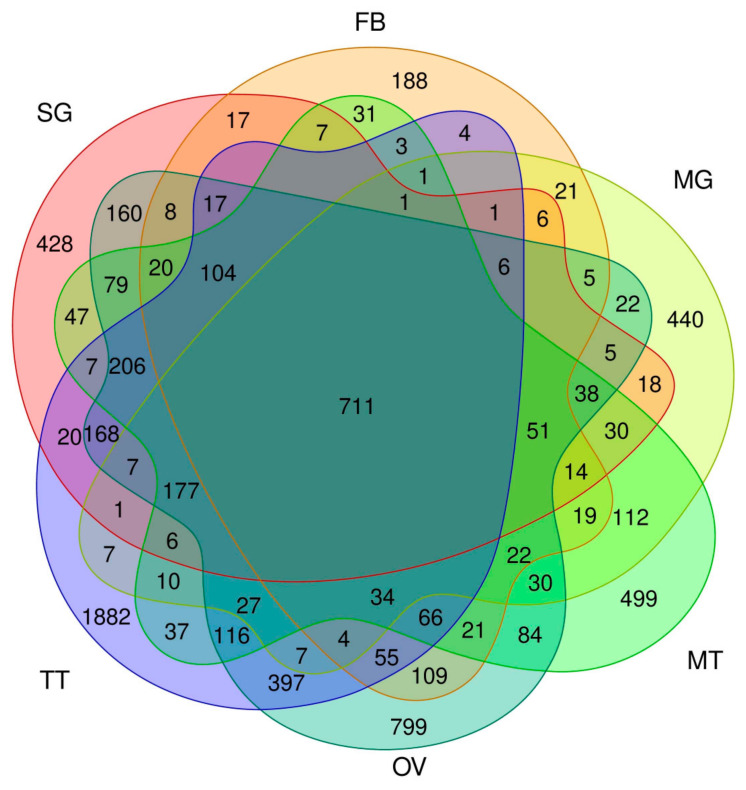
Venn diagram showing the transcripts expressed in each tissue. The number of transcripts with a tpm value > 30 is shown. SG—silk gland; FB—fat body; MG—mid gut; MT—Malpighian tubules; OV—ovary; TT—testis.

**Figure 6:**
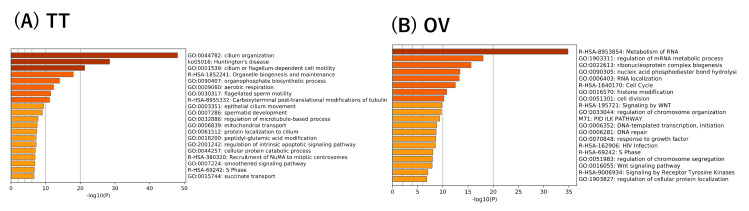
Results of the enrichment analysis by Metascape in the testis (TT) (**A**) and ovary (OV) (**B**). An enrichment analysis was performed using annotation data against the human gene set of the reference transcripts expressed in specific tissues. −log10 (P) represents −log10 (*p*-value). For example, −log10 (*p*) = 5 represents *p*-value = 10^−5^.

**Figure 7:**
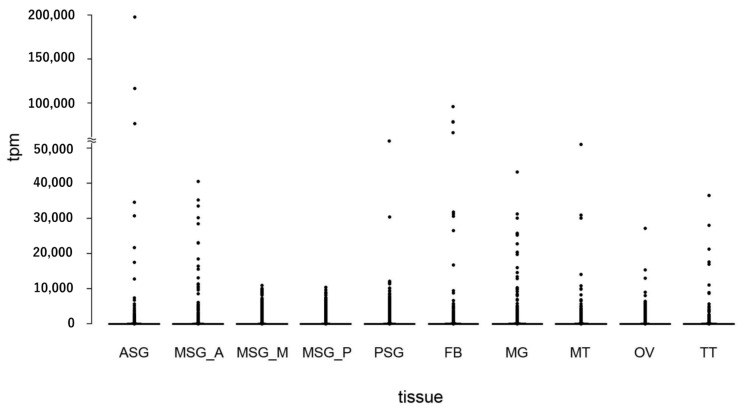
A scatter plot of the transcript expression in each tissue. Each spot shows the tpm value.

**Figure 8:**
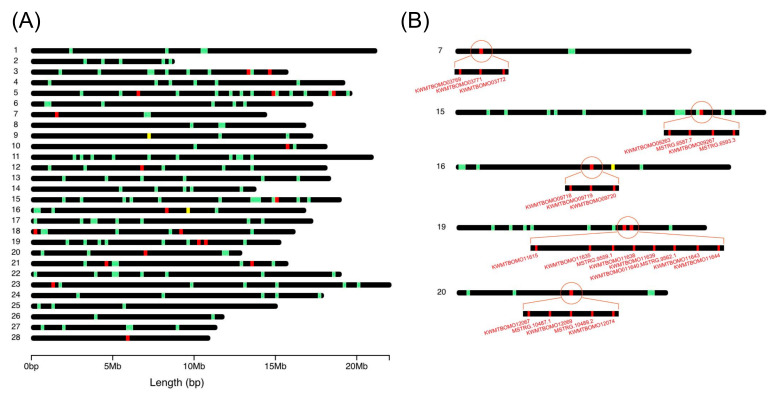
Genomic position of the transcripts strongly expressed in the MG only (red bar), in other than the MG (green bar), and commonly in the MG and other tissues (yellow bar). The top 50 strongly expressed transcripts are shown. The black bar indicates the chromosome. The number at the left side of each chromosome indicates the chromosomal number. (**A**) The genomic positions of all chromosomes. (**B**) The genomic positions of chromosomes 7, 15, 16, 19, and 20, in which tight clusters of strongly expressed transcripts in the MG are present.

Using the data described above, we investigated the transcriptomic expression in detail in each tissue. We focused on transcripts with a tpm value > 30, which accounted for approximately the top 5% of the most strongly expressed transcripts, and regarded such transcripts as being expressed in each tissue. To reveal the whole profile of these strongly expressed transcripts, we investigated which tissues these transcripts were expressed in, and counted the number of transcripts expressed in a single or multiple tissues (Figure 5). We found that 711 transcripts were expressed in all of the tissues (Figure 5), suggesting ubiquitous functions. We also detected transcripts expressed in specific tissues (Figure 5 and Table S5). Among them, transcripts solely expressed in TT were the most abundant (1882), followed by those expressed in OV (799), MT (499), and MG (440) (Figure 5 and Table S5). We also identified transcripts expressed in more than two tissues, such as TT and OV (397; Figure 5). FEA was performed on transcripts with a tissue-restricted expression, and the functional clusters enriched in each tissue were very diversified; for example, transcripts exclusively expressed in the TT were strongly enriched for “cilium organization”, “Huntington’s disease”, and “cilium or flagellum-dependent cell motility” (Figure 6A), whereas “Metabolism of RNA”, “regulation of mRNA metabolic process”, and “ribonucleoprotein complex biogenesis” were enriched in the OV (Figure 6B). Comparisons of the transcript expression levels among the tissues revealed that the expression levels of the strongly expressed transcripts were very high in the ASG (Figure 7). These transcripts comprised fungal protease inhibitors, cuticular protein genes, and others (Table S6). In contrast, the levels of strongly expressed transcripts were lower in the MSG-M/MSG-P (Figure 7 and Table S6). We also investigated the expression profiles of the transcripts strongly expressed in each tissue, and found tissue-restricted expression for these transcripts in the MG, FB, and MSG_A and a ubiquitous expression for those in the other tissues examined (Table S6 and Figure S9). The genomic positions of the tissue-enriched transcripts were examined, and the transcripts strongly expressed in the MG formed tight genomic clusters (Figure 8 and Table S6). We also found clusters for strongly expressed transcripts in other tissues (Table S6 and Figure S10). 

**Figure 9:**
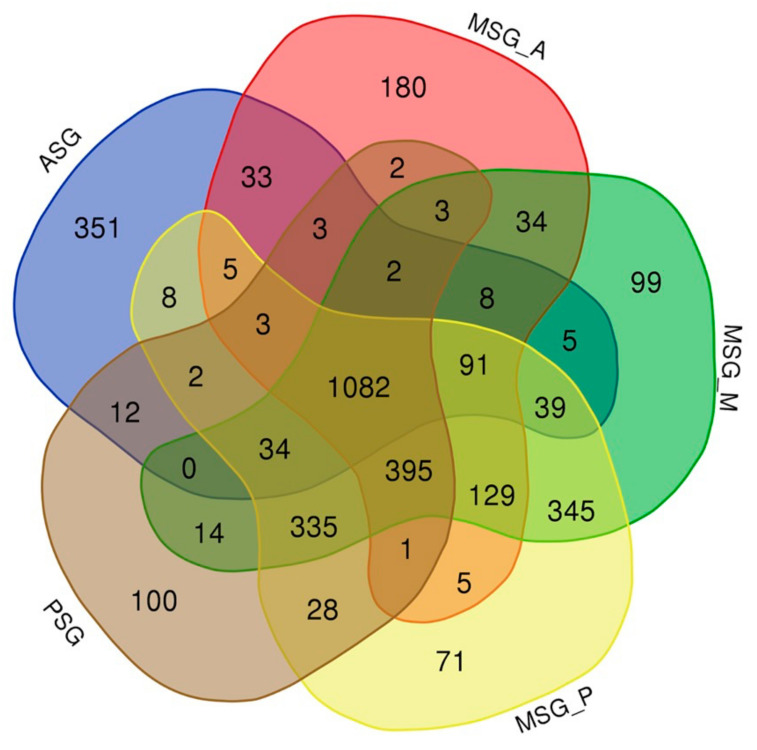
Venn diagram showing the number of transcripts with a tpm value > 30 in each silk gland part.

We then investigated the transcript expression in the SG in more detail. Previous studies revealed that a number of transcripts showed a territory-specific expression in the SG [28,29]. However, the overall transcript expression in each territory remained unclear. Herein, we demonstrated that >1000 transcripts were commonly expressed in all SG subparts, and also that a number of transcripts were expressed in specific territories (Figure 9). They included 351 ASG-restricted, 180 MSG_A-restricted, 99 MSG_M-restricted, 71 MSG_P-restricted, and 100 PSG-restricted transcripts, respectively (Figure 9 and Table S7). Furthermore, we identified transcripts that were commonly expressed in more than two territories (Figure 9). They included transcripts expressed in MSG_M and MSG_P (Figure 9), and combined with the results of HC and the correlation analysis (Figure 4 and Table S4), we speculate that gene expression is highly conserved between MSG_M and MSG_P. This result was supported by the presence of a smaller number of transcripts expressed solely in the MSG_M or MSG_P (Figure 9). We also found that the transcripts that were exclusively expressed in ASG were more abundant than in other territories, and fewer transcripts were commonly expressed in the ASG and other subparts (Figure 9). We speculate that this reflects the functional diversification of the ASG, because the numbers of transcripts exclusively expressed in ASG (351) and those expressed in the MSG and PSG (395) were comparable, suggesting similar diversified functions (Figure 9). This may also be the case for MSG_A, based on the presence of similar characteristics (Figure 9). FEA revealed that the functional clusters enriched in each SG subpart were largely diversified (Figure S11).

### 3.6. Expression Analysis of Transcriptional Factor Genes in the Silkworm

**Figure 10:**
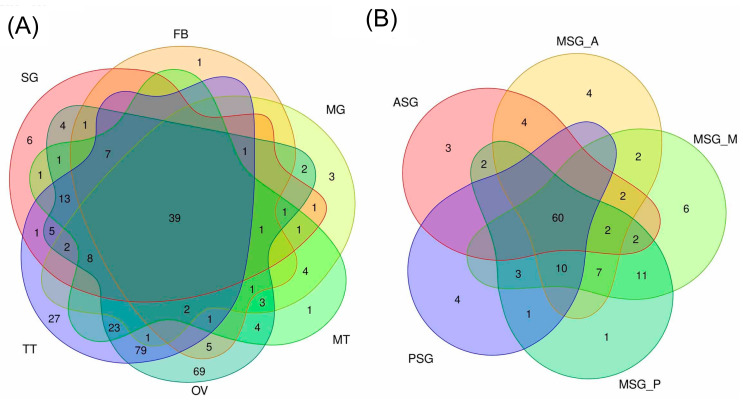
Venn diagrams showing the number of transcriptional factor (TF) transcripts with a tpm value > 5 (**A**) in each tissue and (**B**) in the silk gland subparts.

A transcriptomic analysis is a powerful tool for identifying genes with low levels of expression. TF genes are considered to show low expression levels, even though they have many important functions in developmental, physiological, and other major biological processes. Therefore, an expression analysis of TF genes will contribute to a more detailed understanding of the silkworm biology. Silkworm TF genes have recently been catalogued [37] and we investigated their expression levels in various silkworm tissues using this information. According to the low level of expression of TF genes, herein, we perceived TF transcripts with a tpm value > 5 as those expressed in each tissue. This analysis revealed that a number of TF transcripts were exclusively expressed in the OV and/or TT (Figure 10A, Table S8). These transcripts included KWMTBOMO02002 (traffic jam), KWMTBOMO002212 (mirror), KWMTBOMO01693 (vismay), and KWMTBOMO06584 (Sox100B), all of which play significant roles in gonad morphogenesis, oogenesis, spermatogenesis, as well as TT differentiation in *Drosophila melanogaster* (Table S8) [38,39,40,41]. We also found that KWMTBOMO09369/KWMTBOMO10218, the silkworm counterparts of human GATA4, were expressed in the OV, similar to that in humans (Table S8) [42]. We speculate that these TF genes have conserved functions in a wide variety of organisms, possibly in the development, differentiation, or homeostasis of reproductive tissues.

**Figure 11:**
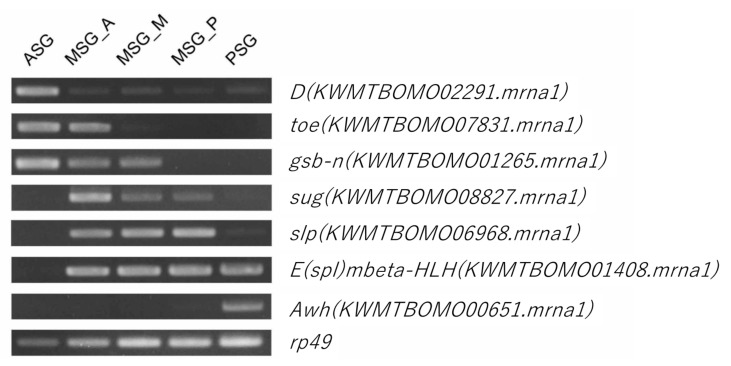
RT-PCR of TF transcripts showing territory-specific expression in the SG.

We also investigated TF gene expression in the SG. Previous studies revealed the central roles of the TF genes forkhead (fkh), Antennapedia (Antp), and Arrowhead (Awh) in the regulation of the silk gene expression [43,44,45,46,47]. Other TF genes that function in the regulation of silk gene expression may also exist because silk genes are strongly expressed and a number of silk genes are expressed in specific territories in the SG (Figure 9). In addition to previously identified TF genes, the expression of novel TF genes, such as those belonging to the High Mobility Group (HMG), Zinc Finger (ZF), Paired box (PAX), or forkhead family, was confined to the SG (Figure 10B and Table 2). The tpm values of these transcripts are shown in Table S8, and we identified TF genes expressed solely in the ASG or MSG_A. Transcripts expressed in more than two subparts were also detected (Table S9). We confirmed their expression in an RT-PCR analysis and the experimental results were consistent with the tpm values (Figure 11). The TF genes identified herein include those with indispensable roles in the development of *D. melanogaster*; for example, Dichaete (D), gooseberry-neuro (gsb-n), and sloppy paired 2 (slp2) are essential for embryonic segmentation [48,49,50], enhancer of split mβ-HLH (E(spl)mβ-HLH) for neurogenesis [51], and twin of eyegone (toe) for eye development [52]. We consider that it is very interesting if these genes have significant roles in the regulation of the silk gene expression, a function that is independent from the developmental regulation, in the silkworm.

## 4. Discussion

In the present study, we performed RNA-seq analysis of multiple larval tissues from the silkworm *B*. *mori*. We established RTD using a recently reported high-quality reference genome assembly [15] and RNA-seq data newly obtained herein. RTD showed marked improvements over GMD, most notably the establishment of a nearly complete structure for *Ser1* (Figures S3 and S4); its full sequence has never been entirely elucidated because of its complexity. Our results indicate that an extensive RNA-seq analysis in combination with high-quality reference genome data provides highly refined gene structures, even for complex genes. The cost of performing a deep sequencing analysis has recently decreased and, thus, it has become affordable for every researcher to conduct not only a short-read RNA-seq analysis, but also long-read genome sequencing, using their own species. The present results are a significant proof-of-concept that highly refined gene structures may be established using a combination of these data, even for non-model organisms. Furthermore, more elaborate gene structures may be constructed using the RNA-seq data derived from other tissues and/or stages.

Herein, we found that a number of transcripts showed a tissue-restricted expression in the silkworm (Figure 5). Among them, transcripts exclusively expressed in the TT were the most abundant (Figure 5). A previous study identified a number of TT-specific genes in the silkworm [12], which is consistent with the present results. The presence of a number of TT-specific genes was also demonstrated in the jewel wasp, *Nasonia vitripennis* [53], indicating that this is a common feature in insects. Recent studies on *Drosophila* revealed that newly emerging genes were strongly biased for expression in the male reproductive system [54]. Therefore, the TT-specific transcripts identified in the present study may have similar traits. This issue may be confirmed by investigating the evolutionary ages of these genes, and, if this is the case, addressing the question of why the TT is a tissue that is permissive for new gene birth, a phenomenon observed not only in insects, but also in vertebrates [55], which will become possible using the silkworm. Another important result obtained from our cross-tissue gene expression analysis is that genes strongly expressed in the MG showed a strong tissue-restricted expression and also formed tight genomic clusters (Figure 8, Table S6, and Figure S9). Comparisons of the sequences of these transcripts revealed that the transcripts in each cluster encoded homological proteins; chr.7 transcripts encoded trypsins, chr.15 juvenile hormone-binding proteins (JHBPs), chr.16 fatty acid-binding proteins, chr.19 actin cytoskeleton-regulatory complex proteins, and chr.20 multiprotein bridging factor 2 (MBF2; Table S6). The predicted amino acid sequences of the transcripts within each cluster annotated with the same functions are similar to each other. Among these genes, a strong expression in the MG has already been demonstrated for *jhbp*s [56], and Trypsin proteins were present in the digestive juices [57]. Homological genes that cluster in the genome are generally considered to have originated via tandem gene duplication, and to the best of our knowledge, few studies have investigated clustered genes that are expressed in the MG. In one case study on *Drosophila*, neutral lipase genes expressed in the MG clustered in the genome and were presumably under positive selection to retain different substrate specificities towards new lipid components of the diet [58]. Based on these findings, the transcripts identified in the present study may also have advantages in the silkworm MG, such as enhancing the activity for digestion and/or xenobiotic detoxification. The present study provides valuable insights into gene evolution and neofunctionalization in insects, which may be validated in more detail in future studies. In addition, the results obtained herein will facilitate the practical application of the silkworm; targeted gene integration into the clusters identified in the present study will enable strong gene expression in the MG, which will contribute to the establishment of strains with valuable properties, such as increased antiviral or antibiotic activities. The latest genome editing technologies should promote the establishment of useful silkworm strains.

Our detailed transcript expression analysis provides fundamental information on the traits of the SG, particularly each subpart. The SG is a tissue that arises from a single embryonic segment and has a long tubular structure [59]. Transcriptomic expression in each SG subpart is largely diversified, as demonstrated herein (Figure 9 and Table S7) and in previous studies [28,29]. The most important result of the present study is that the transcriptomic expression in the ASG was diversified the most within the SG, as demonstrated by the presence of a number of transcripts exclusively expressed in the ASG (351; Figure 9), a number of transcripts expressed both in the MSG and PSG (395; Figure 9), and the location of the ASG at the outermost site in the SG cluster in the HC analysis (Figure 4). We speculate that these results indicate the functional diversification of the ASG, which is consistent with previous findings showing that the ASG functions in silk fiber processing and the MSG/PSG in silk protein production [60]. FEA revealed that functional clusters enriched in the ASG were the “carbohydrate metabolic process” and “transport of small molecules” (Figure S11A), while those enriched in the MSG/PSG were “ribonucleoprotein complex biogenesis” and “translation” (Figure S11B–E), further supporting this concept. In this context, another result showing that the transcript expression in the MSG_A also showed diversification is of interest. We demonstrated that transcripts specifically expressed in the MSG_A were abundant (180; Figure 9), a number of transcripts were commonly expressed in the MSG_M/MSG_P/PSG (335; Figure 9), and the MSG_A was located at the outermost site in the MSG/PSG cluster in the HC analysis (Figure 4). MSG_A is a part of the MSG and functions in the production of the sericin proteins *Ser2* and *Ser3* [35,61], similar to the function of the MSG_M/MSG_P in the production of *Ser1* [32]. Nevertheless, our results indicate that the transcript expression in the MSG_A was more diverse than that in the MSG_M/MSG_P compared with that of the PSG; PSG is the territory that produces fibroin and not sericin [62], whereas the transcript expression appeared to be more conserved between the PSG and MSG_M/MSG_P than between the MSG_A and MSG_M/MSG_P (Figure 4 and Figure 9). These results may be attributed to the presence of a number of transcripts that are strongly and specifically expressed in the MSG_A, including ecdysone oxidase, fatty acid hydroperoxide dehydratase, and other transcripts (Figure 9 and Table S7). We found that transcripts strongly expressed in the MSG_A were enriched for the functional clusters of “metabolism of vitamins and cofactors” and “organic hydroxy compound transport” (Figure S11B), and speculated that these clusters define the biological functions that are unique to this territory. Therefore, our extensive RNA-seq analysis provides fundamental insights into the functions of the SG, as well as its evolution, which has not yet been elucidated in detail. In the *Lepidoptera*, the morphology of the SG is largely diversified among species [63] and the *Saturniidae*, a family that is phylogenetically close to the *Bombycidae*, have MSGs with one territory and no morphological separation [6]. Therefore, the differentiation of the MSG gene expression observed herein may be specific to *B*. *mori* and/or other closely related species. Further studies are needed to clarify whether the differences in the gene expression among species are a driving force that generates diversity in cocoon properties, including shape, size, and physical activity. The TF genes identified in the present study may be one of the key factors responsible for the differences in the gene expression within the SG or among species.

## 5. Conclusions

We carried out RNA-seq analysis on the major larval tissues of the silkworm. Using these data, we successfully improved the gene model greatly as well as clarified gene expression in detail in each tissue. Our result should be a fundamental basis for the further promotion of the silkworm study as well as contribute to the practical application of the insects.

## Figures and Tables

**Table 2:** List of transcription factors expressed in the silk gland (more detailed data was in Table S9). human_gname and fly_gname indicate functional annotations against human and fly genomes, respectively, shown in Data S3.

Transcript ID	Family	Human_Gname	Fly_Gname
KWMTBOMO02291.mrna1	HMG	SOX14	D
KWMTBOMO02290.mrna1	HMG	SOX21	Sox21b
KWMTBOMO07638.mrna1	ETS	SPDEF	Ets98B
KWMTBOMO08935.mrna1	zf-LITAF-like		CG30273
KWMTBOMO08827.mrna1	zf-C2H2	GLIS2	sug
KWMTBOMO10947.mrna1	zf-CCCH	ZFP36L1	Tis11
KWMTBOMO09826.mrna1	zf-C2H2	ZBTB49	Clamp
KWMTBOMO12968.mrna1	Fork_head	FOXN1	jumu
KWMTBOMO15603.mrna1	zf-C2H2	ZNF606	CG9215
KWMTBOMO01121.mrna1	zf-C2H2	NAF1	CG10341
KWMTBOMO00301.mrna1	zf-LITAF-like		CG32280
KWMTBOMO07252.mrna1	zf-C2H2	PRDM10	
KWMTBOMO03284.mrna1	zf-BED		
KWMTBOMO09501.mrna1	TRAM_LAG1_CLN8	CERS5	schlank
KWMTBOMO00651.mrna1	Homeobox	LHX8	Awh
KWMTBOMO07825.mrna1	MYB	SMARCA1	Iswi
KWMTBOMO08651.mrna1	Homeobox	PBX1	exd
KWMTBOMO11294.mrna1	zf-C2H2	ZNF891	CG17328
KWMTBOMO07831.mrna1	PAX	PAX2	toe
KWMTBOMO01266.mrna1	PAX	PAX3	gsb-n
KWMTBOMO02915.mrna1	Fork_head	FOXB1	fd96Ca
KWMTBOMO07945.mrna1	zf-C2H2	OVOL1	ovo
KWMTBOMO01265.mrna1	PAX	PAX3	gsb-n
KWMTBOMO13459.mrna1	Pou	POU3F4	vvl
KWMTBOMO07731.mrna1	zf-LITAF-like	LITAF	CG13510
KWMTBOMO15317.mrna1	TF_bZIP	FOSL1	kay
KWMTBOMO07734.mrna1	zf-LITAF-like		CG13510
KWMTBOMO10990.mrna1	TF_bZIP	ATF3	Atf3
KWMTBOMO07931.mrna1	ETS	ETV6	aop
KWMTBOMO01705.mrna1	zf-C2H2	KLF10	cbt
KWMTBOMO16597.mrna1	zf-C2H2	KLF18	
KWMTBOMO02077.mrna1	zf-C2H2	GFI1B	sens

## Data Availability

The RNA-seq reads supporting the conclusions of the present study are available in the SRA with accession number DRA008737 (the accession number of the RNA-seq data of each sample is shown in Table 1). Reference transcriptome data are available at the Transcriptome Shotgun Assembly (TSA) database under accession IDs ICPK01000001-ICPK01051926, and the Gff file of the transcriptome is available via doi:10.18908/lsdba.nbdc02443-001.V001. The estimated abundance of transcripts is available from the Gene Expression Archive (GEA) in DDBJ under accession ID E-GEAD-315 or from The Life Science Database Archive (doi:10.18908/lsdba.nbdc02443-002.V001).

## Supplementary Materials

Additional data are available in The Life Science Database Archive. The title in the Archive is “KAIKO—Metadata of reference transcriptome data” (http://doi.org/doi:10.18908/lsdba.nbdc02443-000.V001; Accessed on 21 April 2021) and figshare (http://doi.org/doi:10.6084/m9.figshare.c.5333894; Accessed on 28 May 2021). Table S1: Primer sequences used for RT-PCR (http://doi.org/doi:10.6084/m9.figshare.14217308; Accessed on 21 April 2021). Table S2: Tpm values in MSTRG.494.1, MSTRG.649.1-2, and MSTRG.704.1-3 (http://doi.org/doi:10.6084/m9.figshare.14206034; Accessed on 21 April 2021). Table S3: Comparisons of the *sericin-1* amino acid composition elucidated by an amino acid analysis and gene model. Mole% is shown (http://doi.org/doi:10.6084/m9.figshare.14206748; Accessed on 22 April 2021). Table S4: Spearman’s rank correlation coefficient among silk gland territories (http://doi.org/doi:10.6084/m9.figshare.14217071; Accessed on 22 April 2021). Table S5: List of transcripts expressed in specific tissues (http://doi.org/doi:10.6084/m9.figshare.14217206; Accessed on 22 April 2021). Table S6: List of transcripts strongly expressed in each tissue or in the silk gland subparts. The top 50 strongly expressed transcripts are shown (http://doi.org/doi:10.6084/m9.figshare.14217218; Accessed on 22 April 2021). Table S7: List of territory-specific transcripts in the silk gland (http://doi.org/doi:0.6084/m9.figshare.14217242; Accessed on 22 April 2021). Table S8: List of tissue-specific TF genes (http://doi.org/doi:10.6084/m9.figshare.14217272; Accessed on 22 April 2021). Table S9: List of territory-specific TF genes in the silk gland, (http://doi.org/doi:10.6084/m9.figshare.14217296; Accessed on 12 May 2021). Data S1: Predicted amino acid sequences of reference transcriptome (http://doi.org/doi:10.18908/lsdba.nbdc02443-004; Accessed on 21 April 2021). Data S2: Functional annotations of the reference transcriptome (blast against human and *Drosophila* gene sets; http://doi.org/doi:10.18908/lsdba.nbdc02443-003; Accessed on 21 April 2021). Data S3: Functional annotations of the reference transcriptome (blast against the NCBI nr database; http://doi.org/doi:10.6084/m9.figshare.14192741; Accessed on 21 April 2021). Data S4: Expression data of each transcript in multiple tissues (http://doi.org/doi:10.18908/lsdba.nbdc02443-002.V001; Accessed on 2 April 2021). Figure S1: Comparison of gene structures among gene model data, cDNA-based data, and reference transcriptomic data. (A) Locus around KWMTBOMO00087, (B) KWMTBOMO00196, and (C) KWMTBOMO00222. GMD—gene model data; CBD—cDNA-based data; RTD—reference transcriptomic data (http://doi.org/doi:10.6084/m9.figshare.14205785; Accessed on 22 April 2021). Figure S2: Expression of new loci transcripts that did not hit the cDNA-based gene model. Numbers indicate transcripts with a tpm value > 0.01 in each tissue. SG—silk gland (using average tpm values in the five SG subparts); FB—fat body; MG—mid gut; MT—malpighian tubules; OV—ovary; TT—testis (http://doi.org/doi:10.6084/m9.figshare.14217335; Accessed on 22 April 2021). Figure S3: Structure of the *sericin-1* gene. MSTRG.2477.1 has a long exon 6. GMD—gene model data; CBD—cDNA-based data; RTD—reference transcriptomic data (http://doi.org/doi:10.6084/m9.figshare.14206166; Accessed on 22 April 2021). Figure S4: The presumptive full-length amino acid sequence of *sericin-1* deduced by the reference transcriptomic data (MSTRG.2477.1). The orange characters show the amino acids encoded by exon 6 and blue characters by exon 8 (http://doi.org/doi:10.6084/m9.figshare.14206736; Accessed on 22 April 2021). Figure S5: Sequence of the 38-amino acid-based repeat unit encoded by exon 6 and exon 8 in *sericin-1*. Exon 6 comprises 53 repeats and exon 8 comprises 13 repeats (http://doi.org/doi:10.6084/m9.figshare.14212673; Accessed on 22 April 2021). Figure S6: Comparison of the *sericin-3* sequence among different gene models. The amino acid sequences identified in the previous study (NM_001114644; [23]), derived from cDNA-based data (BMgn014348), reference transcriptomic data (MSTRG.2595.1), and gene model data (KWMTBOMO06311), are shown. Amino acids that differ in KWMTBOMO06311 are shown as white letters. Note that a frame shift occurs in the gene model data (http://doi.org/doi:10.6084/m9.figshare.14216927; Accessed on 22 April 2021). Figure S7: Comparison of the *sericin-4* gene structure. The gene structures elucidated by the previous study (*sericin-4*; [24]) and modeled by the gene model data (GMD) and reference transcriptomic data (RTD) are shown (http://doi.org/doi:10.6084/m9.figshare.14216951; Accessed on 22 April 2021). Figure S8: (A) Principal component analysis (PCA) results with expression profiles in 45 samples of transcripts showing a tpm value > 30 in at least one sample. Abbreviations and the numbers of samples are the same as in Figure 4. The X axis and Y axis are the principal components 1 (PC1) and PC2, respectively. (B) Hierarchical clustering of the expression data in 45 samples using all transcript tpm values (http://doi.org/doi:10.6084/m9.figshare.14216993; Accessed on 22 April 2021). Figure S9: A scatter plot of the transcript expression in each tissue. Each spot shows the tpm value. The top ten ranked strongly expressed transcripts in (A) ASG, (B) MSG_A, (C) MSG_M, (D) MSG_P, (E) PSG, (F) FB, (G) MG, (H) MT, (I) OV, and (J) TT are marked in red (http://doi.org/doi:10.6084/m9.figshare.14217227; Accessed on 22 April 2021). Figure S10: Genomic position of the genes strongly expressed in each tissue. In (A), the genes strongly expressed in the ASG only are shown with a red bar, in subparts other than the ASG with a green bar, and those commonly expressed in the ASG and other tissues with a yellow bar. The same applies to MSG_A (B), MSG_M (C), MSG_P (D), PSG (E), FB (F), MT (G), OV (T), and TT (I). (A’) shows the chr. 11 of the ASG and (F’) shows chr. 20 of the FB. Regarding MG, see Figure 8. The top 50 strongly expressed transcripts are shown. The black bar indicates the chromosome, and the number at the left side of each chromosome indicates the chromosomal number (http://doi.org/doi:10.6084/m9.figshare.14217233; Accessed on 22 April 2021). Figure S11: Results of the enrichment analysis for territory-specific transcripts in the silk gland. The number of transcripts used for the analysis is shown in the bracket. −log10 (P) represents −log10 (*p*-value). For example, −log10 (*p*) = 5 represents *p*-value = 10^−5^, (http://doi.org/doi:10.6084/m9.figshare.14217257; Accessed on 22 April 2021).
